# Decoding face categories in diagnostic subregions of primary visual cortex

**DOI:** 10.1111/ejn.12129

**Published:** 2013-02-03

**Authors:** Lucy S Petro, Fraser W Smith, Philippe G Schyns, Lars Muckli

**Affiliations:** 1 Centre for Cognitive Neuroimaging (CCNi), Institute of Neuroscience and Psychology, College of Medical, Veterinary and Life Sciences, University of Glasgow58 Hillhead Street, Glasgow, G12 8QB, UK; 2 School of Psychology, University of GlasgowGlasgow, UK

**Keywords:** diagnostic information, fMRI, multivariate pattern classification, V1

## Abstract

Higher visual areas in the occipitotemporal cortex contain discrete regions for face processing, but it remains unclear if V1 is modulated by top-down influences during face discrimination, and if this is widespread throughout V1 or localized to retinotopic regions processing task-relevant facial features. Employing functional magnetic resonance imaging (fMRI), we mapped the cortical representation of two feature locations that modulate higher visual areas during categorical judgements – the eyes and mouth. Subjects were presented with happy and fearful faces, and we measured the fMRI signal of V1 regions processing the eyes and mouth whilst subjects engaged in gender and expression categorization tasks. In a univariate analysis, we used a region-of-interest-based general linear model approach to reveal changes in activation within these regions as a function of task. We then trained a linear pattern classifier to classify facial expression or gender on the basis of V1 data from ‘eye’ and ‘mouth’ regions, and from the remaining non-diagnostic V1 region. Using multivariate techniques, we show that V1 activity discriminates face categories both in local ‘diagnostic’ and widespread ‘non-diagnostic’ cortical subregions. This indicates that V1 might receive the processed outcome of complex facial feature analysis from other cortical (i.e. fusiform face area, occipital face area) or subcortical areas (amygdala).

## Introduction

Previous studies have found evidence supporting the theory that substantial information is transferred from higher visual areas to V1, even outside the classical receptive field. Electrophysiological recordings in primates reveal that V1 is exposed to considerable feedback modulation (Bullier, 2001; Thiele *et al*., 2009; Self *et al*., 2012), and functional brain imaging experiments show that V1 is involved in cognitive tasks including visual spatial attention (Kanwisher & Wojciulik, 2000; Ress & Heeger, 2003; Watanabe *et al*., 2011), mental tracking (Kaas *et al*., 2010), mental imagery (Slotnick *et al*., 2005), visual expectation (Kastner *et al*., 1999) and visual working memory (Harrison & Tong, 2009). V1 neurons display responses outside the classical receptive field (Angelucci *et al*., 2002; Harrison *et al*., 2007; Muckli & Petro, 2013; Shushruth *et al*., 2013), and modulation in non-stimulated areas by apparent motion along the illusory path (Muckli *et al*., 2005) and by surrounding scene context (Smith & Muckli, 2010).

Top-down influences on early visual cortex during face processing are still relatively unexplored, although a recent study in behaving monkeys revealed that evoked neural population responses in V1 correlate with the perceptual processing of faces (Ayzenshtat *et al*., 2012), reported to be feedback related. This result is supported by anatomical studies showing direct feedback projections from temporal cortices, including the superior temporal sulcus (Rockland & Van Hoesen, 1994), which is involved in processing facial expressions in humans (Haxby *et al*., 2000). The application of multivariate statistics to activation patterns in early visual cortex uncovers rich visual information content, beyond that expected of neurons yielding small receptive fields (Kamitani & Tong, 2005, 2006; Kay *et al*., 2008; Miyawaki *et al*., 2008; Walther *et al*., 2009; Smith & Muckli, 2010; Meyer, 2012). Cortical feedback to early visual areas is implicated in several of these findings, and there is no reason to preclude a contribution during face processing (Ayzenshtat *et al*., 2012). Additional input to early visual areas may arise from amygdala neurons (Vuilleumier *et al*., 2004; Gschwind *et al*., 2012), reported to coordinate responses to biologically salient stimuli such as faces, which are thought to be primarily cortical in nature (Pessoa & Adolphs, 2011).

Using functional magnetic resonance imaging (fMRI), we investigated whether V1 is modulated by task during face processing in subregions responding to two facial features (eyes and mouth). These features task-dependently activate higher visual regions (Gosselin & Schyns, 2001; Schyns *et al*., 2003, 2007, 2009; Smith *et al*., 2004, 2005, 2008, 2009, 2012). In a univariate analysis we explored changes within these ‘eye’ and ‘mouth’ regions-of-interest (ROIs) as a function of task. We used multivariate pattern analysis (MVPA; e.g. Haynes & Rees, 2005; Kamitani & Tong, 2005, 2006; Walther *et al*., 2009; Smith & Muckli, 2010; Chiu *et al*., 2011) to determine if the remaining (‘non-diagnostic’) regions of V1 can decode expression and gender, and we compared this with the decoding performance in each of the target subregions of V1. We show that feature-specific regions of V1 engage in face discriminations in a task-specific manner, and further that the remainder of V1 also contains task-relevant information.

## Materials and methods

### Participants

Nine subjects (21–29 years, five males) with normal vision were screened for potential health risks. All subjects gave written, informed consent, and the experiment was approved by the local ethics committee of the College of Science and Engineering (University of Glasgow) under the project number FIMS00579. The experiment conforms to the Code of Ethics of the World Medical Association (Declaration of Helsinki).

### Stimuli

The same images were used for gender and expression categorization tasks. Face stimuli were grey-scale images of five males and five females taken under standardized illumination, all displaying happy, fearful and neutral expressions. (Neutral was included to maintain a reasonable level of task difficulty, i.e. to minimize subjects performing the task using only one feature, e.g. ‘happy’ or ‘not happy’ using the wide open mouth; see also Smith *et al*., 2008). Stimuli were normalized for location of mouth and eyes, and comply with the Facial Action Coding System (CAFE database; Ekman & Friesen, 1978; unpublished data). Face stimuli spanned 19° × 13° of visual angle; the large size of the images was required in order to obtain clear separation in early visual cortex of the two features of interest. For retinotopic mapping of the eyes and mouth, contrast-reversing checkerboards (4 Hz) were presented in the location at which these features appeared in the face stimuli. The mouth checkerboard spanned 2.8° × 7.2°, and the eye checkerboards 2.8° × 3.6° of visual angle. The vertical distance from the bottom of the eye checkerboard to the top of the mouth checkerboard was 4.9°. The total pixel area of the mouth checkerboard was equal to the summed area of the two eye checkerboards.

We chose to map the eyes and the mouth for two reasons, one methodological and one conceptual. Firstly, we required a clear separation of features in the cortex, achievable by selecting the features with sufficient spatial distance between them. Secondly, behavioural and brain-imaging evidence have shown that eye or mouth information can be selectively extracted from the same image depending on the task. For example, gender is judged using eye information and expressive-or-not by the mouth (Gosselin & Schyns, 2001; Smith *et al*., 2004). Further, we used happy and fearful faces, which typically require mouth and eye information, respectively (Smith *et al*., 2005; Schyns *et al*., 2007). Thus, we had an *a priori* expectation that regions of the cortex representing these features may respond task dependently.

### Design and procedure

#### Face categorization and retinotopic mapping of features

**Fig. 1:**
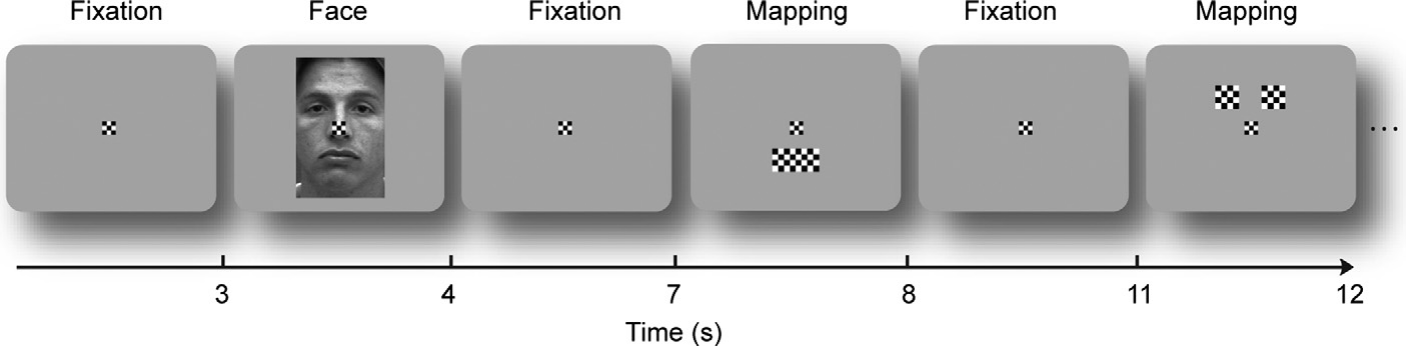
Time line of stimulus sequence.

Prior to scanning, subjects briefly viewed the images to confirm that correct gender and expression classification could be performed. During the rapid event-related fMRI experiment, stimuli were generated using Presentation software (version 10.3; Neurobehavioral Systems) and presented using an MR-compatible binocular goggle system [NordicNeuroLab (NNL), Bergen, Norway; Engström *et al*., 2005]. Eye movements of the right eye were monitored using the NNL Eyetracking Camera, and data collected using a ViewPoint EyeTracker® by Arrington Research. The experiment consisted of presenting one of six different experimental conditions in each run – happy, neutral or fearful face, mapping of eyes or mouth, or fixation baseline. Subjects were instructed to keep fixation on the small central fixation checkerboard (subtending 0.44° × 0.46°) throughout the whole experiment. Faces were centred and normalized for location of features and illumination, and presented at a constant size and view. Face and mapping conditions were presented (randomly ordered) for 1 s, and were followed by 3 s of fixation (Fig. 1). Subjects performed 720 trials (120 per condition) split into six functional runs (each lasting approximately 8.5 min). Runs alternated between expression (three alternative forced choices) and gender categorization (two alternative forced choices) tasks. A button pad was used for response, with the same buttons being used in each task in order that differential patterns of neural activity in V1 are not attributed to motor responses. We expected happy and fearful face images to induce slightly different activation patterns in V1 due to different low-level properties (e.g. higher contrast of the eyes in ‘fear’ or the teeth in ‘happy’). To ensure that activation was not solely driven by these properties, subjects performed both expression and gender tasks on the identical images.

#### Retinotopic mapping of early visual areas

**Fig. 2:**
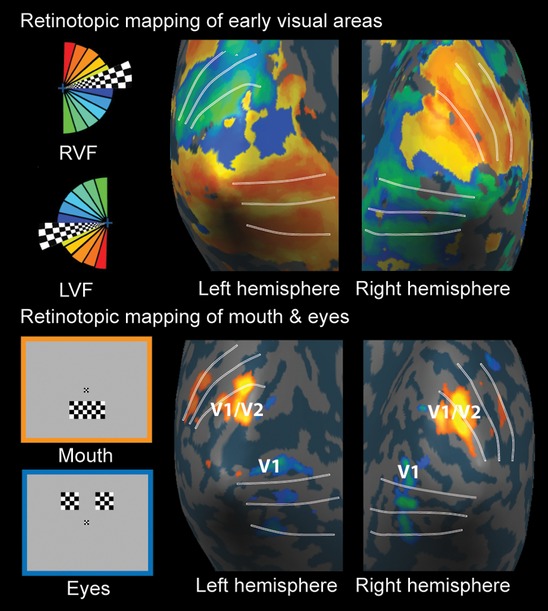
(Upper) Retinotopic mapping of early visual areas using a standard phase-encoded rotating checkerboard, shown for one subject in both hemispheres. The borders between early visual areas are indicated by white lines. (Lower) Feature mapping conditions (left) to identify regions of V1 (right) responding to the eyes (blue/green) and mouth (yellow/orange) of the same subject.

Early visual areas were mapped using a standard phase-encoded polar angle protocol (Sereno *et al*., 1995) using standard parameters employed in our lab (Muckli *et al*., 2005, 2009; Fig. 2).

### MRI procedures

#### Imaging

Imaging was performed at the Centre for Cognitive Neuroimaging, Glasgow, using a 3T Siemens Tim Trio MRI scanner (Siemens, Erlangen, Germany) with a 12-channel head coil. An echo planar imaging sequence was used for parallel imaging [17 slices in an oblique orientation roughly in line with the AC-PC plane (angled around the *x*-axis at −5° on average) to cover visual cortex; repetition time (TR), 1 s; echo time (TE), 30 ms; flip angle (FA), 62°; field of view (FOV), 210 mm; resolution isotropic voxel size 2.5 mm; and gap thickness, 10% (0.25 mm), PACE motion correction; iPAT factor 2]. In addition, T1-weighted anatomical scans were acquired for all subjects (TR, 2 s; TE, 4.38 ms; FA, 15°; FOV, 240 mm, isotropic voxel size, 1 mm^3^).

#### Data analysis

Analysis was performed using BrainVoyager software 1.10.4 (BrainInnovation, http://www.brainvoyager.com) and Matlab 2007b (Mathsworks). The first two volumes of each run were discarded due to T1 saturation effects. Standard pre-processing was as follows – slice scan time correction was performed using sync interpolation based on the TR of 1000 ms and on the ascending, interleaved order of slice scanning. Standard three-dimensional motion correction to adjust for head movements was performed as well as linear-trend removal and temporal high-pass filtering at 0.006 Hz. After alignment with the anatomical scan, all individual datasets were transformed into Talairach space (Talairach & Tournoux, 1988).

#### Retinotopic mapping

A cross-correlation analysis was used for the retinotopic mapping experiment. We used the predicted haemodynamic signal time course for the first 1/8th of a stimulation cycle (32 volumes/4 volumes per predictor) and shifted this reference function slowly clockwise in time (four volumes corresponding to 45° visual angle).

#### Cortical surface reconstruction and patch-of-interest (POI) definition

High-resolution anatomical scans were used to reconstruct surfaces of both cortical hemispheres for all nine subjects (Kriegeskorte & Goebel, 2001). Inhomogeneity correction of signal intensity was followed by segmentation of the white and grey matter border. Functional data were projected onto the inflated hemispheres allowing the borders between early visual areas to be identified (Fig. 2). Checkerboard mapping of mouth and eye regions were used to identify three POIs: (i) ‘mouth’ in V1/V2; (ii) ‘eyes’ in V1; (iii) rest-of-V1 region (V1 without mouth and eye regions). In order to create a rest-of-V1 POI that was not immediately adjacent to the eye and mouth POIs, we increased the size of the patches by lowering the threshold of eye and mouth maps, and subtracted these patches from the entire V1 patch. We ensured that the rest-of-V1 region and entire V1 regions included only vertices sampled in the main experimental runs by intersecting these functionally constrained regions with a map defined from the set of functionally responsive voxels – all faces minus baseline, threshold (*P* < 0.0001, corrected).

#### Eye movements

Data were linearly detrended per run and transformed into units of degrees of visual angle before being classified as a saccade if a succession of samples had a radius > 1.5° of visual angle for a duration of 150 ms (Weigelt *et al*., 2007). Mean vertical and horizontal fixation locations were compared across tasks to reveal no significant differences.

#### General linear model (GLM) deconvolution – univariate analysis

We used a GLM deconvolution approach (20 predictors per condition excluding baseline) to estimate blood oxygen level-dependent (BOLD) response amplitudes to faces in ‘mouth’ and ‘eye’ POIs, independently for gender and expression tasks. Differences in the beta values (parameter estimates in the GLM analysis) were tested for significance using anovas. Contrasts of mouth and eye checkerboard mapping conditions were used to define two non-overlapping ROIs in each hemisphere, in individual subjects – a ‘mouth’ region in dorsal V1/V2 and an ‘eye’ region in ventral V1. Thresholds were kept above 3.2, but were slightly adjusted individually in order to get the most optimal separation of feature regions for subjects 1–9 (S1–9) as follows: S1, *t*_3002_ > ± 3.70; S2, *t*_3002_ > ± 3.82; S3, *t*_3002_ > ± 3.61; S4, *t*_3002_ > ± 3.62; S5, *t*_3002_ > ± 3.78; S6, *t*_3002_ > ± 3.78; S7, *t*_3002_ > ± 3.29; all *P* < 0.0003 correcting for multiple comparisons using a false discovery rate correction of 0.01. However, for two subjects thresholds had to be lowered to *t*_3002_ > ± 2.27 (S8) and *t*_3002_ > ± 2.07 (S9) in order to obtain comparable ROIs.

#### MVPA

For each participant we applied a GLM to estimate single trial response amplitudes (Kay *et al*., 2008; Kriegeskorte *et al*., 2008; Smith & Muckli, 2010) for each vertex time course, independently per run and POI. The design matrix pertaining to single trial response estimation consisted of as many columns (predictors) as trials (plus one for mean confound), coding stimulus presentation. This was then convolved with a standard 2 gamma model of the haemodynamic response function. These single trial response estimates (beta weights), taken according to the corresponding subregion of V1 (see POIs i–iii above), were the input to the pattern classifier. We trained a linear pattern classifier [Support Vector Machine (SVM)], independently per participant per region of V1, to learn the mapping between a set of multivariate brain observations and the corresponding expression label (happy or fear). We then tested the ability of the classifier to generalize to an independent set of test data. Thus, we trained the classifiers with a set of single trial brain activity patterns and tested the classifiers either on independent ‘single trials’ or on the ‘average’ brain activity pattern for each expression in the independent set of test data. In order to control for the different number of vertices present within each POI, a factor that could influence classifier performance, we used a sub-sampling approach comparable to Smith & Muckli (2010). This involved randomly selecting a subset of vertices – i.e. sampling 30 times for each of several different vertex set sizes (1 : 10 : 160, giving 15 different set sizes), and building independent classifiers for each random selection of vertices at each vertex set size. To estimate the performance of our classifiers we used a leave one run out cross-validation procedure (see Kamitani & Tong, 2005; Walther *et al*., 2009; Smith & Muckli, 2010) – that is, the classifier was trained on *n*−1 runs and tested on the independent *n*th run (repeated for the *n* different possible partitions of the runs in this scheme).

To estimate the amount of task-related top-down influence, independent classifiers were constructed for trials in which the subjects were engaged in an expression classification task (explicitly doing the same classification happy/fear) or in a gender classification task (where the happy/fear classification might only be made implicitly). We report across subject average performance and use one-sample *t*-tests (one-tailed) to test whether average performance, for the maximal number of vertices, was significantly greater than chance (50%). The linear SVM was implemented using the LIBSVM toolbox (Chang & Lin, 2001), with default parameters (notably C = 1). Note that the beta weights of each vertex were normalized in the training data within a range of −1 to 1 prior to input to the SVM. Test data were independently normalized using the same parameters (max, min, range) as the training data.

## Results

### Behaviour

In alternating runs of fMRI recordings, subjects discriminated either emotional content or gender of the same 30 images (three emotions, ten identities, two genders).

#### Reaction time

**Fig. 3:**
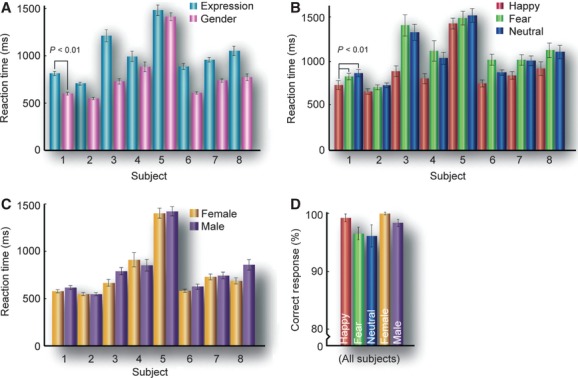
(A) Per subject average reaction times during expression (3AFC) and gender (2AFC) tasks (asterisks show significance to *P* < 0.01 across subjects). (B) Average reaction times to happy, fearful and neutral faces during the expression task. (C) Average reaction times to female and male faces during the gender task. (D) Categorization accuracy for happy, fearful, neutral, male and female judgements (error bars state 1 SE).

Data contributing to behavioural analysis included eight of the nine subjects due to technical reasons. Subjects were faster to respond during the gender task than during the expression task (anova of correct trials: *F*_1,7_ = 23.6, *P* = 0.001). Within tasks, subjects were significantly faster to respond to happy faces than to fearful and neutral faces (*F*_2,14_ = 14.8, *P* = 0.0004), and equally fast to categorize female and male faces (*F*_1,7_ = 2.9, *P* = 0.12; Fig. 3A–C).

#### Accuracy

During fMRI scans, accuracy across subjects was 98.7, 96.0 and 95.8% for ‘happy’, ‘fear’ and ‘neutral’, respectively, in the expression task (anova: *F*_2,14_ = 1.77, *P* = 0.21), and 99.5% and 97.9% for females and males, respectively, in the gender task (*F*_1,7_ = 1.88, *P* = 0.22; Fig. 3D).

### ‘Mouth’ and ‘eye’ POIs – univariate analysis

We localized the cortical representation of the eyes and mouth using checkerboards covering each respective area. The contrast of checkerboard mapping conditions was used to define non-overlapping POIs in each hemisphere in individual subjects – a ‘mouth’ region in dorsal V1/V2 and an ‘eye’ region in ventral V1. From within ‘mouth’ and ‘eye’ regions we extracted the average deconvolved BOLD responses to face stimuli during both tasks. Tasks alternated between runs but the face stimuli remained identical. We subjected the beta values to three-way anovas in two different ways – firstly taking the individually adjusted peak value (between 5 and 7 s); and secondly averaging across time points 3–9 s.

### Expression classification – peak time point

A three-way anova of the peak BOLD response of classification (happy/fear), task (expression/gender) and region (eyes/mouth) revealed no main effects, but a significant interaction of classification and region (*F*_1,8_ = 38.3, *P* = 0.0003).

### Gender classification – peak time point

A three-way anova of classification (male/female), task (expression/gender) and region (eyes/mouth) revealed no main effects, but a significant interaction of classification and task (*F*_1,8_ = 8.260, *P* = 0.0207).

### Expression classification 3–9 s

Averaging beta across time points 3–9 s, a three-way anova of classification (happy/fear), task (expression/gender) and region (eyes/mouth) revealed no main effects, but significant interactions between classification and region (*F*_1,8_ = 10.2, *P* = 0.01) and between task and region (*F*_1,8_ = 11.4, *P* = 0.009).

### Gender classification 3–9 s

A three-way anova of classification (male/female), task (expression/gender) and region (eyes/mouth) revealed a significant main effect of task (*F*_1,8_ = 5.624, *P* = 0.04), and a significant interaction between classification and task (*F*_1,8_ = 12.1, *P* = 0.008).

### Fixed effects

**Fig. 4:**
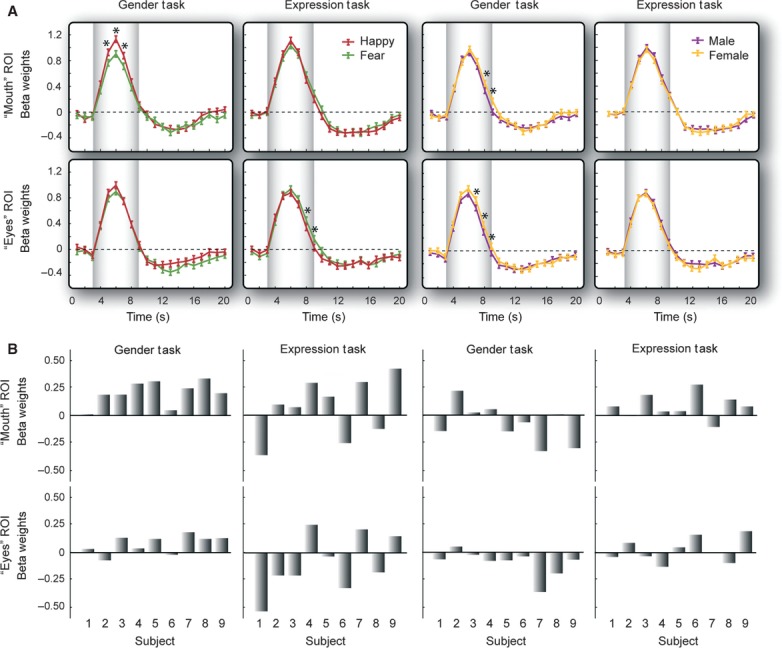
(A) Deconvolved blood oxygen level-dependent (BOLD) signal time courses to happy and fearful faces in eye and mouth patches-of-interest (POIs) during expression and gender tasks across subjects, and deconvolved BOLD signal time courses to male and female faces in eye and mouth POIs during expression and gender tasks, across subjects. Contrasts between happy and fearful faces, and between male and female faces, were tested for significance: (i) for the peak (see text); (ii) collapsed across 3–9 s (see text); and (iii) at individual time points in a general linear model (GLM) with only one predictor per stimulus condition and many confounds (one per participant per run, see asterisks, all passing a Holm–Bonferroni correction). Error bars report standard errors between subjects. (B) Individual subject data of the difference in peak beta values (happy minus fear) and (male minus female).

For a more detailed examination of time points, we also performed a fixed effect analysis, testing individual time points from 3 to 9 s. For this, we ran a GLM with only one predictor per stimulus condition and many confounds (one per participant per run). This revealed differential effects (over the peak of the BOLD signal) of expression (happy > fear) in the ‘mouth’ ROI when judging gender, and in the ‘eyes’ ROI when judging expression (fear > happy, over later time points; Fig. 4A). We also observed an increased response to female over male faces, only at later time points (for these effects, see asterisks in Fig. 4A; all passing a Holm–Bonferroni correction that controls for the family-wise error rate). The differences in peak beta values between happy and fearful faces, and female and male faces, in all ROIs during both tasks can be seen in Fig. 4B for individual subjects.

The deconvolution analysis allows an independent interpretation of time points, as they are estimated individually, but we did not have any a priori hypothesis about different time points and cannot draw firm conclusions from this result. Although speculative, the variance we observed in the time at which differences occurred could be indicative of additional cognitive modules, for example integrating information from a different facial location, itself related to the unfolding of top-down inputs over time. This remains to be tested.

### POI-based MVPA in V1

**Fig. 5:**
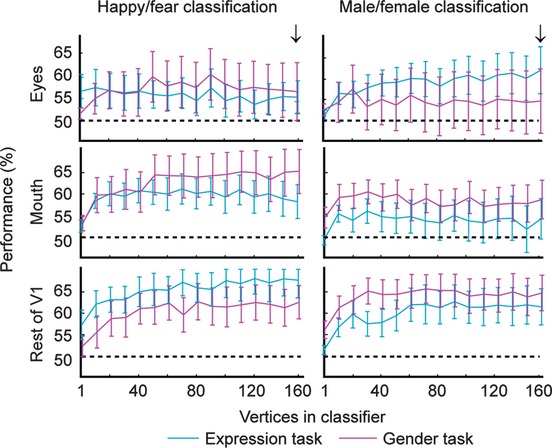
Multivariate pattern classification analysis (MVPA). Percentage performance for classifying facial expression (happy or fear, left) or gender (male, female, right) from activity patterns extracted from mouth, eye and rest of V1 patches-of-interest (POIs), during expression (blue) and gender (pink) tasks. Performance is predicted on averaged data. The arrows show performance for maximum number of voxels sampled (160). Error bars represent 1 SEM.

**Fig. 6:**
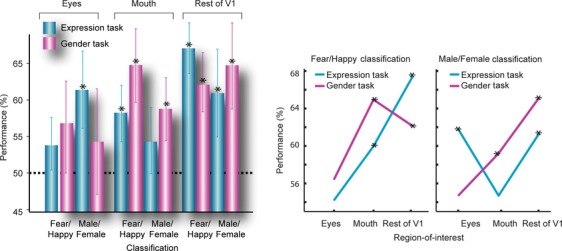
Multivariate pattern classification analysis (MVPA) shown both as a bar graph and line plot (to visualize the interaction, SEM same as bar plot, not shown). Percentage performance of MVPA classification computed on averaged data for classifying expression and gender of faces during expression and gender tasks, for the maximal number of vertices (160, see arrow in Fig. 5; asterisks reveal significance above chance of 50%, *P* < 0.05).

We next addressed, with MVPA, the contribution that the remainder of V1 has in discriminating expression and gender during the two tasks, in comparison to ‘eye’ and ‘mouth’ regions. To this end, we defined a third POI, from the remainder of V1, i.e. not processing the eyes or mouth. Figure 5 and Table 1 show classifier performance as a function of the number of vertices (maximum 160) entering the analysis, when trained to discriminate either expression or gender, for each V1 subregion and task (sub-sampling was used to equate the number of vertices across different V1 subregions). By way of summary, Fig. 6 displays the classifier performance for the maximum number of vertices (arrows in Fig. 5). The classification of happy and fear was significantly above chance in the mouth (performance = 57.96%, *t*_8_ = 2.01, *P* = 0.04) and rest-of-V1 (performance = 67.47%, *t*_8_ = 4.25, *P* = 0.001) POIs during the expression task, and interestingly even when the explicit judgement was gender (performance = 64.94%, *t*_8_ = 2.86, *P* = 0.01 and 62.1%, *t*_8_ = 3.11, *P* = 0.007, in the ‘mouth’ and ‘rest-of-V1’, respectively). In contrast, the classification of gender was significantly above chance in the ‘eyes’ when the task was expression discrimination (performance = 61.79%, *t*_8_ = 2.17, *P* = 0.03), and in the ‘mouth’ when the task was gender discrimination (performance = 59.20%, *t*_8_ = 2.05, *P* = 0.04). Comparable to expression classification, gender classification was highly significant in the rest-of-V1, during both tasks (performance = 61.3%, *t*_8_ = 2.68, *P* = 0.01 and 65.12%, *t*_8_ = 3.68, *P* = 0.003, in expression and gender tasks, respectively). The classifier performance values were submitted to a three-way anova of task (expression or gender), region (eyes, mouth or rest-of-V1) and classification (expression or gender), and revealed a significant effect of region (*F*_1,8_ = 4.89, *P* = 0.02); overall decoding performance was higher in the rest of V1 than either of the diagnostic patches.

**Table 1:** Average (upper) and single-trial (lower) classifier performance (%) in decoding either expression or gender during both tasks, within the cortical representation of the eyes, mouth and remaining V1

	Eyes (%)	Mouth (%)	Rest of V1 (%)
Expression task			
Happy/fear	54.20, *t*_8_ = 1.410, *P* = 0.098	57.96, *t*_8_ = 2.005, *P* = 0.040	67.47, *t*_8_ = 4.253, *P* = 0.001
	54.26, *t*_8_ = 2.050, *P* = 0.037	54.47, *t*_8_ = 2.811, *P* = 0.011	55.39, *t*_8_ = 3.998, *P* = 0.002
Male/female	61.79, *t*_8_ = 2.174, *P* = 0.031	54.69, *t*_8_ = 0.999, *P* = 0.174	61.30, *t*_8_ = 2.681, *P* = 0.014
	52.94, *t*_8_ = 2.380, *P* = 0.022	52.18, *t*_8_ = 1.451, *P* = 0.092	53.50, *t*_8_ = 2.571, *P* = 0.017
Happy/fear/neutral	37.00, *t*_8_ = 0.822, *P* = 0.217	37.90, *t*_8_ = 1.286, *P* = 0.117	42.84, *t*_8_ = 3.850, *P* = 0.024
	35.26, *t*_8_ = 1.323, *P* = 0.112	36.52, *t*_8_ = 2.297, *P* = 0.025	36.93, *t*_8_ = 6.527, *P* = 0.0001
Gender task			
Happy/fear	56.42, *t*_8_ = 0.950, *P* = 0.185	64.94, *t*_8_ = 2.856, *P* = 0.011	62.10, *t*_8_ = 3.106, *P* = 0.007
	52.65, *t*_8_ = 1.848, *P* = 0.051	54.88, *t*_8_ = 2.457, *P* = 0.020	53.97, *t*_8_ = 3.355, *P* = 0.005
Male/female	54.69, *t*_8_ = 0.632, *P* = 0.273	59.20, *t*_8_ = 2.046, *P* = 0.038	65.12, *t*_8_ = 3.678, *P* = 0.003
	51.28, *t*_8_ = 0.933, *P* = 0.189	52.29, *t*_8_ = 1.804, *P* = 0.054	54.08, *t*_8_ = 4.233, *P* = 0.001
Happy/fear/neutral	36.30, *t*_8_ = 0.817, *P* = 0.219	45.56, *t*_8_ = 3.462, *P* = 0.004	39.01, *t*_8_ = 2.038, *P* = 0.038
	34.91, *t*_8_ = 1.453, *P* = 0.092	37.02, *t*_8_ = 2.947, *P* = 0.009	35.06, *t*_8_ = 2.506, *P* = 0.018

One initial motivation for the study was to investigate whether cortical regions processing the features that are most important for a specific categorization task also exhibit the greatest classification information in their activation profile. From previous behavioural research it is known that gender discrimination relies on the processing of the eyes and the detection of a happy expression on processing the smiling mouth. One could therefore expect that there is differential activation in the eye region for male/female classification especially during the gender task, and in the mouth region for happy/fear classification during the expression task. However, there is no indication in our data that supports this hypothesis (see Discussion). The pattern classifier analysis demonstrates that the rest of V1 has a significant contribution to facial expression and gender discrimination (in line with the hypothesis of distributed feedback to V1). In summary, the data show that each subregion of V1 contains important information for discriminating face categories.

## Discussion

Behavioural studies have shown that facial features are extracted task-dependently, for example, eye information is typically used to diagnose gender (Gosselin & Schyns, 2001). Moreover, signals in distributed visual areas are modulated by task-dependent facial feature use, as shown using electroencephalography, magnetoencephalography and fMRI (Smith *et al*., 2004, 2008, 2009; Schyns *et al*., 2007, 2009). In light of findings showing that V1 is susceptible to higher cognitive functions and that rich stimulus information can be decoded from V1 activation profiles (Kamitani & Tong, 2005, 2006; Kay *et al*., 2008; Miyawaki *et al*., 2008; Walther *et al*., 2009; Smith & Muckli, 2010; Meyer, 2012; Muckli & Petro, 2013), we investigated if V1 also contains complex information about facial features, i.e. does V1 contain facial features in a task-dependent manner as higher visual areas do? Specifically, we took advantage of the retinotopic organization of V1 by spatially mapping the visual field coordinates of individual facial features into cortical coordinates. Statistically significant multivariate pattern classification was observed both within these ‘feature’ regions and also in the remaining V1 (responding to the rest of the face). To our knowledge, this is the first study to observe task effects in retinotopically discrete regions of primary visual cortex responding to facial features; however, our data do not offer a straightforward interpretation. To illustrate, we observed the following task effects – in the ‘eye’ region, gender was only decoded during the expression task and not during the gender task; and vice versa in the ‘mouth’ region. Expression, on the other hand, was only ever decoded in the ‘mouth’ region, but not in the ‘eye’ region. Lastly, to our surprise, we observed higher classification of expression and gender in the remaining V1, during both tasks.

Gender decoding in early visual areas has been hinted at in previous studies (trend toward significance; Kaul *et al*., 2011), and anatomical connectivity studies using diffusion tensor imaging support the notion of top-down or recurrent feedback effects via long-range fibre tracts from face areas and the amygdala to early visual cortex (directly or via the occipital face area; Gschwind *et al*., 2012). Given the findings of this latter study, and the task effects that we observed, we are inclined to consider our data in the context of top-down modulation by higher visual areas. Functionally, this modulation could transfer task effects in higher visual areas (Chiu *et al*., 2011) to V1, where the high-resolution spatial map acts as a foundation upon which top-down influences improve stimulus discriminations by targeting early stages of processing (Ahissar & Hochstein, 2002).

Our findings raise at least five questions: (i) How are task-relevant facial features available at the level of V1? (ii) How do task requirements influence V1 processing? (iii) What is the involvement of cortical or subcortical top-down effects and recurrent feedback? (iv) How do our findings relate to studies investigating top-down processing using different techniques? (v) What do our findings suggest about the spatial precision of top-down processing?

### How are task-relevant facial features available at the level of V1?

Task instructions were processed at the beginning of each experimental run after verbal directions, and task effects cannot be explained by feedforward visual processing as identical images were used in both tasks. We suggest that task effects are processed in auditory, multi-modal and central executive areas, likely engaging face-sensitive areas, which may then exert top-down influences to early visual areas. To be explicit, by top-down influence we refer to how high-level categorizations task-dependently modulate internal representations (Chiu *et al*., 2011). In the context of our V1 data, this cognitive process could be reflected in differential baseline levels prior to stimulation, or differential activation subsequent to stimulation. We differentiate here between the cognitive term ‘top-down’ and the anatomical term ‘feedback’. By feedback we refer to recurrent neuronal input to V1 that is not projected from the lateral geniculate nucleus (i.e. extrastriate cortex, pulvinar, amygdala). We discuss below how feedback might be contributing to our data, without differentiating between task-related and non task-related feedback. Task-related (top-down) modulation may or may not be carried by recurrent feedback (i.e. be reflected in baseline activity).

### How do task requirements influence V1 processing?

To our surprise, the gender task did not lead to higher classifier information in the eye region and the expression task did not enhance information in the mouth region, which could be hypothesized based on the idea of diagnostic information (Gosselin & Schyns, 2001). One possible explanation in line with the idea of diagnosticity is that features most relevant for the task are channelled through to higher areas for processing so, for the gender discrimination, the relevant eye region (Gosselin & Schyns, 2001) might be activated in recurrent loops leading to sustained activity that is not differential to male and female images (i.e. ceiling effects). The non-relevant task dimension (emotion information) might still trigger differential feedforward effects (i.e. happy faces vs. fearful faces) that can lead to differential activation patterns in the mouth region. In a predictive coding framework, it could be conceptualized that internal models for gender prototypes are recurrently processed during the gender task leading to maximal activation but no differential activation for this dimension. The non-task-relevant dimension (expression) may lead to surprise (prediction error) responses. Likewise, during an expression task, which may require more mouth information (Gosselin & Schyns, 2001), enhanced activation could reflect ceiling effects and therefore no difference in BOLD signal. This *post hoc* explanation may justify why the classifier performance can be better for differences in the other task than the one that is currently dominant. Our explanation of the direction of task effects is speculative and requires more specific testing.

At this point we would like to acknowledge the possible contribution of low-level features. In a recent study of face viewpoint invariance, V1 was shown to respond sensitively to low-level similarities in a study about view-invariance (Kietzmann *et al*., 2012), and at present we cannot entirely rule out this explanation. Low-level stimulus features, however, cannot account for the task effects as 30 identical stimuli were used in both tasks. Moreover, we and others (Williams *et al*., 2008) have shown in non-stimulated regions of V1, complex contextual effects independently of certain low-level features (even in non-stimulated regions of V1; Smith & Muckli, 2010).

### What is the involvement of cortical or subcortical top-down effects and recurrent feedback?

Task-dependent top-down modulation to V1 can arise from cortical and subcortical areas directly or indirectly connected to V1. This could occur prior to stimulus processing (baseline activity) or in response to stimulus processing (including recurrent feedback). Candidate regions are likely to be recurrently connected to V1 (Felleman & Van Essen, 1991; Clavagnier *et al*., 2005), but we cannot differentiate between top-down modulation and recurrent feedback.

In the case of expression categorization, input to V1 might be relayed through the subcortical superior colliculus–pulvinar–amaygdala pathway (Vuilleumier *et al*., 2004; see Pessoa & Adolphs, 2011 for review). Electrophysiology has shown that the monkey pulvinar responds to facial expression (Maior *et al*., 2010). The pulvinar is known to gate activity to V1 (Purushothaman *et al*., 2012). The human amygdala has been shown to respond to faces and face parts (Rutishauser *et al*., 2011). In the macaque, input to V1 from the amygdala has been shown to originate from the basal nucleus (Freese & Amaral, 2005).

Studies of prosopagnosia (Rossion, 2008) suggest a direct pathway from early visual areas to the right fusiform face area. As mentioned, diffusion tensor imaging studies suggest connectivity between the early visual areas and the occipital face area (Gschwind *et al*., 2012). Whether or not these connections are recurrent and extend to V1, and what functional role they may play remains an interesting question. Recurrent feedback connections from temporal areas may guide face-selective occipital areas to extract fine-grained features (Gauthier *et al*., 2000; Rossion *et al*., 2003), which could logically include V1 given that it contains high spatial resolution information. Although it remains to be tested, it is conceivable that features are extracted in higher cortical or subcortical areas (which are specialized for face processing) and sent back to V1 where they contribute to the V1 BOLD signal, which is especially susceptible to feedback (Muckli, 2010; Schmidt *et al*., 2011; Watanabe *et al*., 2011; Cardoso *et al*., 2012).

### How do our findings relate to studies investigating top-down processing using different techniques?

Inactivation studies using transcranial magnetic stimulation (TMS) in humans (de Graaf *et al*., 2012) or cortical cooling in animal experiments (Schmidt *et al*., 2011) might be able to provide further details about the nature of top-down influences to the processing of complex information in V1. Face information and grating information are similarly affected by TMS pulses between 50 and 100 ms after stimulus onset, indicating recurrent processing in the visual cortex during the first 100 ms. For face images, this TMS interference remains noticeable for longer integrating windows (110–130 ms), indicating longer recurrent processing of face information relative to grating information.

A recent study in behaving monkeys using voltage-sensitive dye imaging demonstrated a biphasic response profile in V1, the second phase corresponding to high-level perceptual processing of the face stimulus, achieved via feedback activation of the primary visual cortex (Ayzenshtat *et al*., 2012). The temporal resolution granted by fMRI does not allow us to draw the same conclusions as this study. However, it is feasible that we are observing task effects due to a secondary temporal response profile in V1 carried by feedback (subsequent to activation in higher face-selective cortex).

### What do our findings suggest about the spatial precision of top-down processing?

Activation profiles of localized subregions of V1 can provide valuable information about the nature of top-down influences, as this activation might either enhance the processing of local (e.g. eyes or mouth), global (head shape) or distributed diagnostic features (Miellet *et al*., 2011). We found evidence supporting globally distributed information across V1, and in a task-dependent manner. Distributed feedback might enhance categorization mechanisms (i.e. by sharpening cortical representations; Hohwy, 2012; Kok *et al*., 2012), change global filter properties (i.e. spatial frequency) or provide contextual information (i.e. relevant for predictive coding; Bar, 2007; Peelen *et al*., 2009; Clark, 2012; Muckli *et al*., 2013). In line with our data, neuroanatomically, feedback is believed to be spatially spread; in higher cortical regions neurons have larger receptive fields and feedback from higher cortical areas is believed to have the same divergent spread as the feedforward connections are convergent (Salin & Bullier, 1995; Cavanaugh *et al*., 2002; Wandell & Smirnakis, 2009). Some of our own previous results support the idea that top-down influences can spread and fan-out to various non-stimulated regions of V1 (Muckli *et al*., 2005; Smith & Muckli, 2010; see also Williams *et al*., 2008; Muckli & Petro, 2013). Classifier performance is highest in the rest of V1, whereas behavioural studies have shown that it is harder to judge expression or gender without eye or mouth information (Gosselin & Schyns, 2001). We believe that the pattern of decoding observed in the rest of V1, which is highly suggestive of a task interaction, is indicative of top-down or recurrent feedback effects that may spread over a greater surface area of the cortex. However, future studies will be necessary to fully understand the complex interplay of top-down and recurrent feedback effects together with bottom-up stimulus processing in the rest of the V1 region.

In conclusion, V1 processes complex face-related information both within and outside the feedforward regions that process diagnostic information. Although the precise function of this top-down modulation remains unknown, we take this as evidence that information is back-projected in a task-related and spatially distributed manner to a larger region of V1 during face processing than the small diagnostic regions that may be driving higher visual areas. We hypothesize that the task-related activation profiles detected in V1 arise from higher visual face-selective cortical areas, with likely subcortical contributions arising from reciprocal connections to the amygdala. Investigating activation with high temporal resolution in functionally defined higher visual areas and subcortical structures concurrently with early visual areas would be a compelling future extension to this work (demanding high temporal resolution fMRI in humans or non-human primate electrophysiological recordings), and central to increasing our understanding of visual information processing in general.

## Abbreviations

BOLD: blood oxygen level-dependent
FA: flip angle
fMRI: functional magnetic resonance imaging
FOV: field of view
GLM: general linear model
MVPA: multivariate pattern analysis
POI: patch-of-interest
ROI: region-of-interest
SVM: Support Vector Machine
TE: echo time
TMS: transcranial magnetic stimulation
TR: repetition time
